# Ameliorative Effect of Gum Acacia on Hookah Smoke-Induced Testicular Impairment in Mice

**DOI:** 10.3390/biom10050762

**Published:** 2020-05-13

**Authors:** Badreldin H. Ali, Suhail Al-Salam, Khalid A. Al Balushi, Mohammed Al Za’abi, Sirin A. Adham, Sumaya Beegam, Priya Yuvaraju, Priyadarsini Manoj, Abderrahim Nemmar

**Affiliations:** 1 Department of Pharmacology and Clinical Pharmacy, College of Medicine and Health Sciences, Sultan Qaboos University, Al-Khod, Muscat 123, Oman; alibadreldin@hotmail.com (B.H.A.); kbalushi@nu.edu.om (K.A.A.B.); zaabi@squ.edu.om (M.A.Z.); priyadarsinimanoj@gmail.com (P.M.); 2 Department of Pathology, College of Medicine and Health Sciences, United Arab Emirates University, Al Ain 17666, UAE; suhaila@uaeu.ac.ae; 3 Department of Biology, College of Science, Sultan Qaboos University, Al-Khod, Muscat 123, Oman; sadham@squ.edu.om; 4 Department of Physiology, College of Medicine and Health Sciences, United Arab Emirates University, Al Ain 17666, UAE; sumayab@uaeu.ac.ae (S.B.); priyay@uaeu.ac.ae (P.Y.); 5 Zayed Center for Health Sciences, United Arab Emirates University, Al-Ain 17666, UAE

**Keywords:** gum arabic, inflammation, oxidative stress, testes, reproductive hormones, hookah smoke, StAR

## Abstract

We investigated some reproductive actions of hookah smoke (HS) exposure (30 min/day, for 30 days) in male mice, and the possible mitigative effect of the prebiotic agent gum acacia (GA) thereon. Control mice were air-exposed (AE). Twenty-four hours after the last exposure, the levels of some plasma reproductive hormones, biochemical markers of inflammation, oxidative and nitrosative stress and testicular histopathology were assessed. The urinary level of cotinine, a major nicotine metabolite, was also measured. HS exposure induced significant decreases in testosterone, estradiol, luteinizing hormone, and androgen binding protein, as well as glutathione reductase activity and levels of nitrite and total nitrite. Plasma inhibin B, alkaline phosphatase, lipopolysaccharide binding protein, uric acid, lactate dehydrogenase, lipid peroxidation, 8-oxo-2’-deoxyguanosine, and cytochrome C were significantly increased following HS exposure. In testicular homogenate, nuclear factor-κB (NF-ĸB), nuclear factor erythroid 2–related factor 2 (Nrf2), interleukin- 6 (IL-6), interleukin-1β (IL-1β), transforming growth factor-β1(TGF- β1), and tumor necrosis factor-α (TNF- α) were all significantly elevated, and the steroidogenic acute regulatory protein (StAR) significantly decreased. Histopathologically, there was slight impairment and disorganization of spermatogenesis. Urinary cotinine concentration was elevated significantly in the HS-exposed group compared with the air-exposed group. GA co-administration mitigated the adverse actions of HS measured. In conclusion, daily exposure to HS at the above dose induced adverse actions on the reproductive system of male mice. GA co-administration significantly mitigated these effects by reducing the inflammation, oxidative and nitrosative stress, via a mechanism involving Nrf2, and reduction of StAR expression.

## 1. Introduction

Hookah smoking (HS) (also termed, water pipe, hubble-bubble, *qalyân*, *lulava*, and *shisha*) is a type of smoking that dates back to the 15th century in India and ancient Persia. It is commonly used in the Middle East, and has recently become a popular leisure pastime among young people in Western countries, especially school and college students [1,2,3,4]. The tobacco in HS is sweetened with either fruit flavors or molasses sugar to make it more aromatic and attractive than cigarette smoke (CS). The tobacco in HS is exposed to strong heat from burning charcoal, and the resultant smoke is equal to or even more toxic than CS [5]. Furthermore, the pattern of addiction to water pipe smoking is very similar to that of CS, and cessation rates do not exceed 28% of previous users having stopped smoking [6].

The pathophysiological actions of CS on the respiratory and other systems have been extensively investigated [7]. However, much less has been reported on HS [8], and there is a common and fallacious viewpoint among tobacco users that HS is safer than CS [9].

We recently studied some biochemical and pathophysiological aspects of the various deleterious actions of exposure of mice to HS on the pulmonary, cardiovascular, and male reproductive systems [10,11,12]. Further, we also tested the effectiveness of nootkatone, an 5’ adenosine monophosphate–activated protein kinase (AMPK) activator isolated from grapefruit, in mitigating these actions [13,14].

Gum acacia (GA) is an established prebiotic dietary fiber [15] that has many drug, cosmetic, industrial, and food uses, and a good safety profile [16]. Several studies have shown that GA possesses strong actions against inflammation, oxidative stress, and apoptosis [17]. In some recent clinical trials GA has also been shown to be effective and safe in patients with rheumatoid arthritis [18], obesity [19], and dyslipidemia among patients with sickle cell anemia [20]. However, as far as we know, the possible mitigative action of GA on HS-induced testicular damage in mice has not been previously communicated, and this is the subject of this study.

## 2. Materials and Methods

### 2.1. Animals

Male C57BL/6 mice (9–10 weeks old, initially weighting about 25 g) were provided by the Animal Facility of the United Arab Emirates University (UAEU) (ERA_2017_5625, 21 June 2017), and kept in a room at a temperature of 22 ± 2 °C, relative humidity of about 60%, with a 12-h light–dark cycle (lights on at 6:00 a.m.), with free access to standard pelleted diet. All the procedures involving animals and their care were carried out as previously described [13], and the approval to conduct the experiments was obtained from the Animal Research Ethics Committee of the UAE University.

### 2.2. Exposure to HS

This procedure has been described fully in previous publications [13,21,22]. After seven days of acclimatization to the housing conditions, the mice were divided randomly into four equal groups (*n* = 8 per group): air-exposed (AE, control), HS-exposed (30 min each day for 30 consecutive days), GA-treated (15%, *w*/*v*, in the drinking water for 30 days), and exposed to HS and concomitantly given GA (same dose as above). The doses of HS and GA were chosen from earlier research carried out in our laboratory [13,23].

### 2.3. Blood Collection and Tissue Preparation

Following the last session of exposure to either HS or air, each mouse was anesthetized using sodium pentobarbital (45 mg/kg) intraperitoneal injection and blood was withdrawn from the inferior vena cava and placed in tubes with the anticoagulant ethylenediaminetetraacetic acid (4% EDTA). The blood obtained was spun at 4 °C for 15 min at 900× *g*, and the plasma harvested was kept at −80 °C pending analysis. The animals were then sacrificed with an overdose of anesthesia. The testes from each mouse were removed, rinsed with ice-cold phosphate buffer saline (PBS, pH 7.4) and weighed. The left and half of the right testes were immediately frozen at −80 °C to await biochemical and molecular analyses. The other half of the right testis was placed in Bouin’s fluid for an hour, and then in 10% formalin and processed for histopathology as described earlier [23].

### 2.4. Biochemical Estimations in Plasma and Urine

Measurements of testosterone, luteinizing hormone (LH), estradiol, androgen-binding protein (ABP), inhibin B, and lactate dehydrogenase (LDH) were carried out by ELISA techniques, and uric acid (UA) and alkaline phosphatase (ALP) by an autoanalyzer (BS-120, Mindray, Shenzhen, China), as reported earlier [14]. Additionally, we measured the plasma concentration of lipopolysaccharide-binding protein (LBP) using an ELISA kit from CUSABIO (Hubei, Wuhan, China). The urinary level of cotinine was assayed by an ELISA kit from Creative Diagnostics (Shirley, NY, USA).

### 2.5. Biochemical Measurements in Testicular Homogenates

The testes were dissected out, and the right testis from each mouse was washed with ice-cold normal saline, weighed, and minced. Part of the testis was homogenized (to give a 10% *w*/*v* homogenate) in cold potassium phosphate buffer (pH 7.4, 0.05 M), and the homogenate was spun down at 900× *g* for 10 min at 4 °C. The following analytes were measured by either ELISA or spectrophotometrically, as reported earlier: Lipid peroxidation as malondialdehyde (MDA), glutathione reductase (GR), total nitric oxide (NO) and nitrite/nitrate, and protein [14]. A portion of the homogenate was spun down further at 4000× *g* for 30 min at 4 °C, and the supernatant collected was used to measure cytochrome C. The remaining supernatant was centrifuged at 10,000× *g* for 20 min at 4 °C, and the supernatant obtained was further centrifuged at 12,000× *g* for 20 min at 4 °C to obtain the post-mitochondrial supernatant, which was used for 8-hydroxy-2-deoxyguanosine (8-OHdG), as before [14].

### 2.6. Histopathology of Testicular Sections

From the fixed testicular tissue, four μm sections were prepared from paraffin blocks, stained with hematoxylin and eosin, and examined under a light microscope by a histopathologist (S.A.-S.) unaware of the treatments, as described fully before [14].

### 2.7. Superoxide Dismutase (SOD) Immunohistochemistry

Five-micrometer testicular sections were prepared and mounted on aminopropyltriethoxysilane (APES) coated slides. Following dewaxing with xylene and rehydrating with graded concentrations of alcohol, slides were placed in a 0.01 M citrate buffer solution (pH 6.0) and pretreatment procedures to unmask the antigens were carried out in a water bath at 95 °C for 30 min. Then, sections were treated with peroxidase block for 30 min followed by protein block for another 30 min. Sections were then incubated at room temperature with anti-superoxide dismutase (SOD) rabbit polyclonal antibody (1:100) from Abcam (Cambridge, UK), for one hour. After conjugation with primary antibodies, sections were incubated at room temperature with a secondary antibody (EnVision™ Detection System, DAKO, Agilent, Santa Clara, CA, USA) for 20 min followed by addition of 3,3’-Diaminobenzidine (DAB) chromogen (EnVision™ Detection System, DAKO, Agilent, Santa Clara, CA, USA) and counterstaining carried out with hematoxylin. Appropriate positive controls were used. For negative control, the primary antibody was not added to sections. Positive and negative controls were included in every batch of slides that were stained (not shown in figures).

### 2.8. StAR Western Blotting

The method used here was essentially as reported by [24]. Expression bands of steroidogenic acute regulatory protein (StAR) protein were visualized using the ChemiDoc™ touch imaging system (Bio-Rad, Hercules, CA, USA). Glyceraldehyde 3-phosphate dehydrogenase (GAPDH) was used for normalization, and the densitometry was calculated using the image lab software version 5.2.1 (Bio-Rad, Hercules, CA, USA).

### 2.9. Drugs, Chemicals, and Kits

GA was bought from Sigma-Aldrich (St. Louis, MO, USA). The rest of the chemicals and kits were of the highest grade available, and their sources are mentioned above.

### 2.10. Statistical Analysis

All values reported represent means ± standard error of the mean (SEM). Statistical significance was evaluated by one-way analysis of variance (ANOVA) followed by Bonferroni’s multiple comparison tests using GraphPad Prism software, version 5.03 (San Diego, CA, USA). The *p* < 0.05 was considered significant.

## 3. Results

### 3.1. Effect of HS on Body and Testicular Weights

As shown in Table 1, the 30-day exposure to HS resulted in nonsignificant reduction in the body weight of mice, when compared with AE-exposed mice. GA treatment alone showed a slight nonsignificant body weight reduction, in comparison to the controls. Compared with HS-exposed mice, GA-treated mice and concomitantly treated with HS exposure, showed a slight, nonsignificant increase in body weight.

### 3.2. Effect of HS on the Levels of Some Plasma Hormones, Inhibin B, and Androgen-Binding Protein

As depicted in Table 2, the plasma levels of testosterone, estradiol, and LH were significantly lowered in mice exposed to HS, compared with mice that were exposed to normal air (*p* < 0.05). In mice exposed to HS, there was a significant elevation in inhibin B level, while ABP was slightly but significantly reduced, when compared with mice treated with AE. GA treatment alone did not show any difference when compared with the AE-exposed mice. Simultaneous treatment with HS and GA significantly ameliorated the elevations in the concentrations of these analytes (*p* < 0.05).

### 3.3. Effect of HS on the Levels of Urine Cotinine

As shown in Table 3, the urinary level of cotinine in HS-treated mice was significantly increased, by 156%, when compared with AE-exposed animals. Concomitant treatment with HS and GA significantly reduced the level to 57%, when compared with HS-exposed mice.

### 3.4. Effect of HS on the Concentrations of Some Plasma Analytes

**Figure 1:**
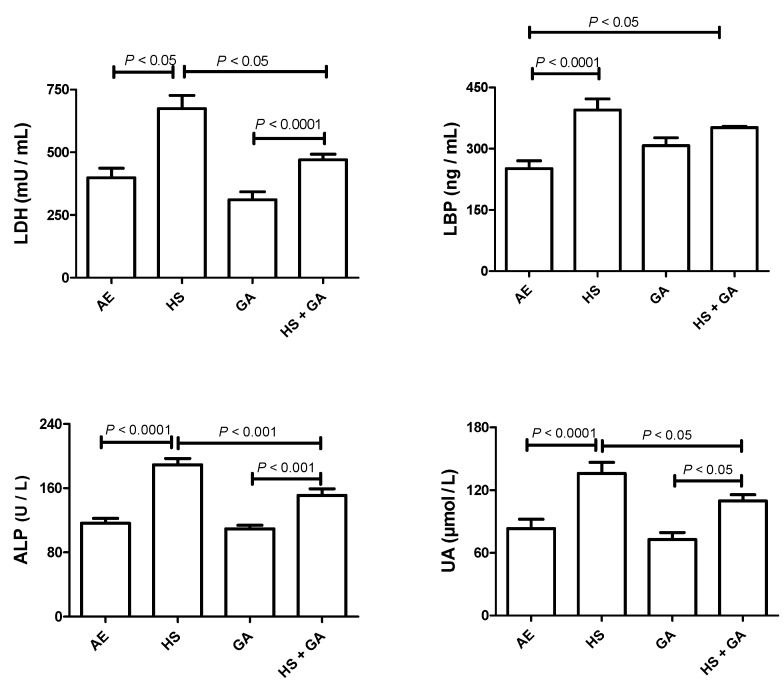
The plasma levels of lactate dehydrogenase (LDH), lipopolysaccharide-binding protein (LBP), alkaline phosphatase (ALP), and uric acid (UA) in mice exposed to normal air (AE), and hookah smoke (HS, 30 min/day) or treated with gum acacia (GA, 15% *w*/*v*) with or without exposure to HS. Each vertical column with bar represents the mean ± SEM (from 8 mice in each group). The *p* < 0.05 was considered significant.

As shown in Figure 1, the levels of plasma ALP, LBP, LDH, and UA were significantly elevated by HS exposure (*p* < 0.05). GA treatment alone did not show any difference when compared with the controls. Co-administration of GA with HS significantly mitigated the HS-induced increase in plasma levels of ALP, LDH and UA, but not LBP.

### 3.5. Effect of HS on Some Cytokines in the Testis Homogenate

**Figure 2:**
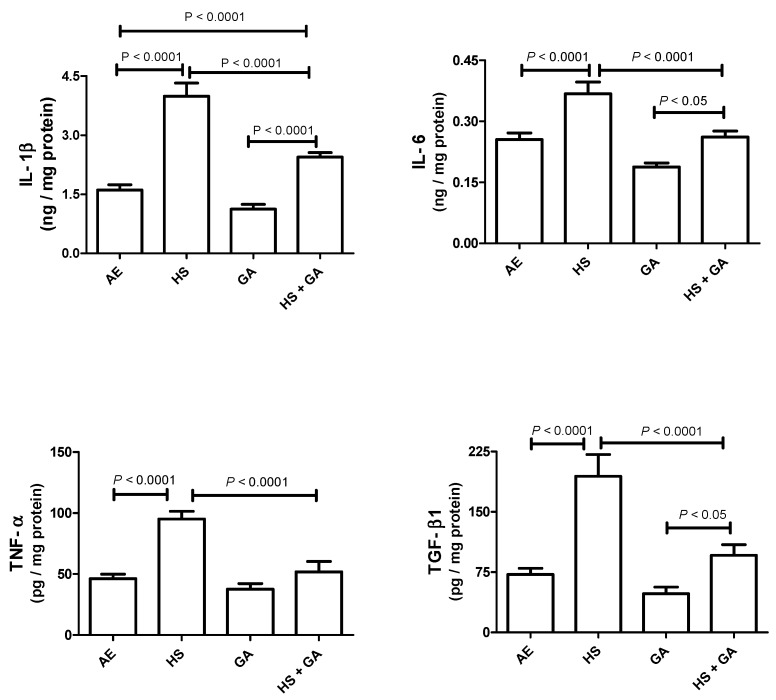
The testicular homogenate concentrations of interleukin 1-beta (IL-1β), interleukin-6 (IL-6), tumor necrosis factor (TNF-α), and transforming growth factor-beta1 (TGF-β1) in mice exposed to normal air (AE) and hookah smoke (HS, 30 min/day) or treated with gum acacia (GA, 15% *w*/*v*) with or without exposure to HS. Each vertical column with bar represents the mean ± SEM (*n* = 8). The *p* < 0.05 was considered significant.

Figure 2 shows that HS exposure significantly raised the concentrations of IL-1β, IL-6, TNF-α and TGF-β1 (*p* < 0.05). GA-treated mice showed slight and nonsignificant reduction in these analytes, when compared with AE-exposed mice. Concomitant treatment with HS and GA significantly ameliorated the elevations in the concentrations of these analytes (*p* < 0.05).

### 3.6. Effect of HS on Some Antioxidants in the Testis Homogenate

**Figure 3:**
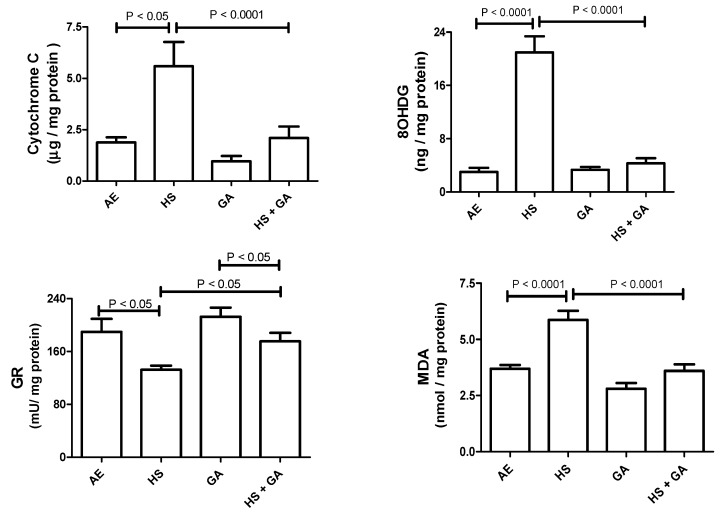
The testicular homogenate concentrations of cytochrome C, 8-oxo-2′-deoxyguanosine (8-OHdG), glutathione reductase (GR), and malondialdehyde (MDA) in mice exposed to normal air (AE) and hookah smoke (HS, 30 min/day) or treated with gum acacia (GA, 15% *w*/*v*) with or without exposure to HS. Each vertical column with bar represents the mean ± SEM (*n* = 8). A *p* < 0.05 was considered significant.

As depicted in Figure 3, mice exposed to HS showed a significant elevation in testis homogenate concentration of cytochrome C, 8-OHdG, and MDA, and a significant decrease of GR. There were no significant differences between the AE- and GA-treated mice in any of the measurements. Concomitant treatment with HS and GA significantly mitigated the elevations in the concentrations of these analytes.

### 3.7. Effect of HS on Nitrosative Stress in the Testis of HS-Exposed Mice

**Figure 4:**
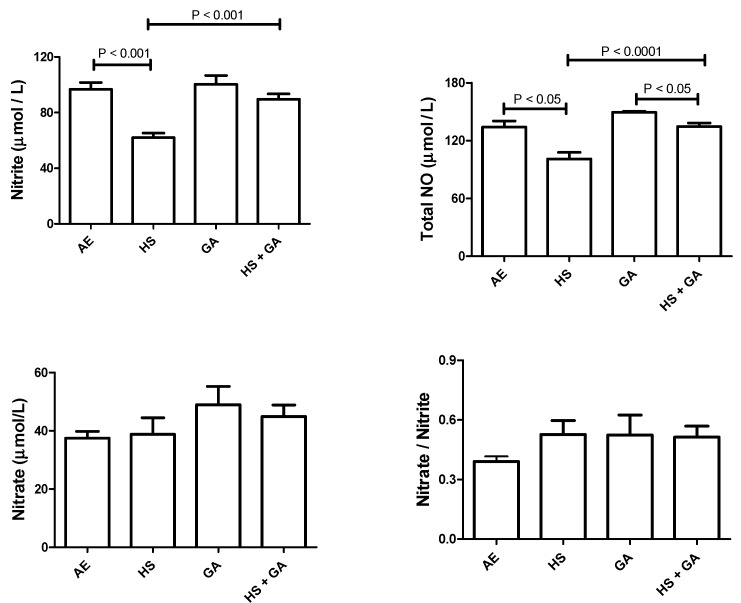
The levels of nitrite, nitrate, total nitric oxide, and nitrate/nitrite in testicular homogenates of mice exposed to normal air (AE) and hookah smoke (HS, 30 min/day) or GA 15% *w* /*v*) with or without exposure to HS. Each vertical column with bar represents the mean ± SEM (*n* = 8). The *p* < 0.05 was considered significant.

Figure 4 show that HS exposure significantly reduced nitrite and total nitric oxide levels (*p* < 0.05). However, the nitrate and nitrate/nitrate levels showed no significant difference when compared with AE-exposed mice. Concomitant administration of GA with HS exposure significantly increased the nitrite and total nitrite level (*p* < 0.05).

### 3.8. Effect of HS on NF-ĸB p65 and Nrf2 in Testicular Homogenate

**Figure 5:**
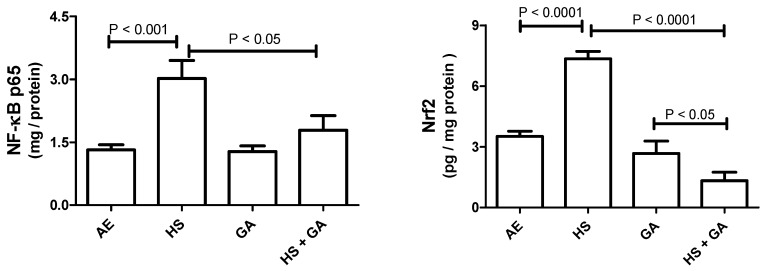
The testicular homogenate concentrations of nuclear factor kappa B (NF-kB) and nuclear factor erythroid 2-related factor-2 (Nrf2) in mice exposed to normal air (AE) and hookah smoke (HS, 30 min/day) or treated with gum acacia (GA, 15% *w*/*v*) with or without exposure to HS. Each vertical column with bar represents the mean ± SEM (*n* = 8). The *p* < 0.05 was considered significant.

As shown in Figure 5, mice exposed to HS showed a significant increase in testis homogenate concentration of NF-ĸB p65 and Nrf2, when compared with AE-exposed mice. GA treatment alone did not show any difference when compared with the controls. However, GA significantly mitigated the HS-induced elevations in the concentrations of these analytes when given concomitantly with HS.

### 3.9. Light Microscopic Histopathology

**Figure 6:**
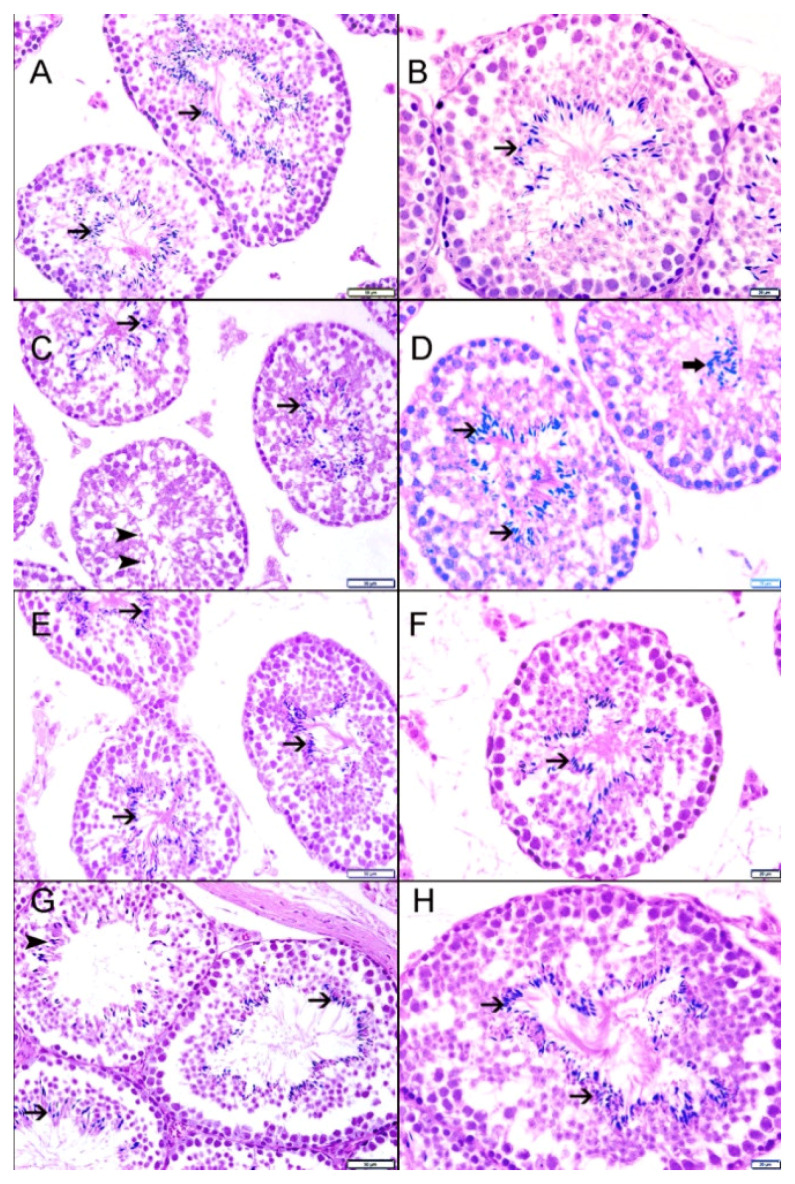
Representative histopathological micrographs, stained with hematoxylin and eosin (H&E), of testicular sections from mice exposed to air (AE), hookah smoke (HS), gum acacia (GA), and HS + GA groups. (**A**,**B**) Representative sections from testes of control group (AE) showing well-maintained spermatogenesis with spermatozoa (arrow) in seminiferous tubules. (**C**) Representative sections from testes of HS-treated group showing well-maintained spermatogenesis with spermatozoa (arrow) in seminiferous tubules; however, some of the tubules showed no spermatozoa but spermatids in seminiferous tubules (arrowhead). (**D**) Representative sections from testes of HS-treated group showing well-maintained spermatogenesis with spermatozoa (arrow) in seminiferous tubules; however, some of the tubules showed disorganized spermatogenesis with accumulation of spermatozoa in the lumen of seminiferous tubules. (**E**,**F**) Representative sections from testes of AE + GA-treated group showing well-maintained spermatogenesis with spermatozoa (arrow) in seminiferous tubules. (**G**,**H**) Representative sections from testes of HS + GA-treated group showing well-maintained spermatogenesis with spermatozoa (arrow) in seminiferous tubules. Some tubules show fewer spermatozoa (arrowhead). Scale bar in images A, C, E, and G = 50 µm. Scale bar in images B, D, F, and H = 20 µm.

Figure 6 and Table 4 show the histopathological pictures and assessment of the testes in the four groups.

In the testicular sections of the control mice, there was complete spermatogenesis (score 10), many spermatozoa present, but disorganized spermatogenesis (score 9), and only a few spermatozoa (score 8) in 51%, 17%, and 20%, of the sections, respectively (Table 4). Spermatogenesis was affected in some of the seminiferous tubules, and in 12% of them there were no spermatozoa, whereas there was block differentiation at the spermatocyte (2%) and spermatid levels (10%). The overall mean *testicular* biopsy score (MTBS) in the control group was 8.979.

In the HS-exposed group, scores of 10, 9, and 8 were obtained for 39%, 17%, and 21%, respectively (Table 4). Spermatogenesis was affected in some of the seminiferous tubules, 23% of the tubules showed no spermatozoa, whereas there was block differentiation at the spermatocyte level in 15%, and at the spermatid level in 8% (Table 4). The overall MTBS in this group was 8.418.

In sections from the GA-treated group, scores of 10, 9, and 8 were obtained for 64%, 13%, and 10%, respectively (Table 4). Spermatogenesis was affected in some of the seminiferous tubules, 13% of the tubules showed no spermatozoa, whereas there was block differentiation at the spermatocyte level in 2%, and at the spermatid level in 11% (Table 4). The overall MTBS in this group was 9.183.

In sections from the group treated with HS + GA, scores of 10, 9, and 8 were obtained for 59%, 12%, and 17%, respectively (Table 4). Spermatogenesis was affected in some of the seminiferous tubules, 12% of the tubules had no spermatozoa, whereas there was block differentiation at the spermatocyte level in 1%, and at the spermatid level in 11% (Table 4). The overall MTBS score in the HS + GA group was 8.94.

### 3.10. Effect of HS Exposure on Testicular SOD Immunohistochemistry

**Figure 7:**
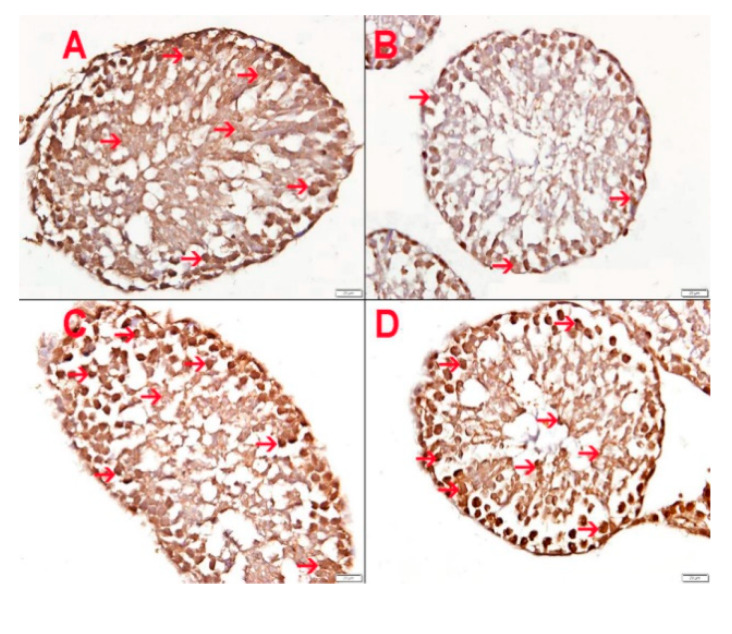
Showing superoxide dismutase (SOD) expression in germ cells within seminiferous tubules. (**A**) Control group exposed to air showing normal expression of SOD in germ cells within the seminiferous tubules. (**B**) Hookah smoke (HS)-exposed group showing a lower expression of SOD by germ cells within the seminiferous tubules. (**C**) GA-treated group showing normal expression of SOD in germ cells within the seminiferous tubules. (**D**) HS-exposed group given GA showed almost normal expression of SOD by germ cells within seminiferous tubules. Scale bar in images A–D = 20 µm.

The expression of SOD in germ cells inside the seminiferous tubules in the control and treated mice is depicted in Figure 7. The control group (A) and the GA-treated group (C) showed normal expression of SOD in germ cells within the seminiferous tubules. The HS-exposed group (B) showed a lower expression of SOD by germ cells within the seminiferous tubules. The HS-exposed group concomitantly treated by GA (D) showed almost normal expression of SOD by germ cells within the seminiferous tubules.

### 3.11. Effect of HS Exposure on Testicular StAR Protein

**Figure 8:**
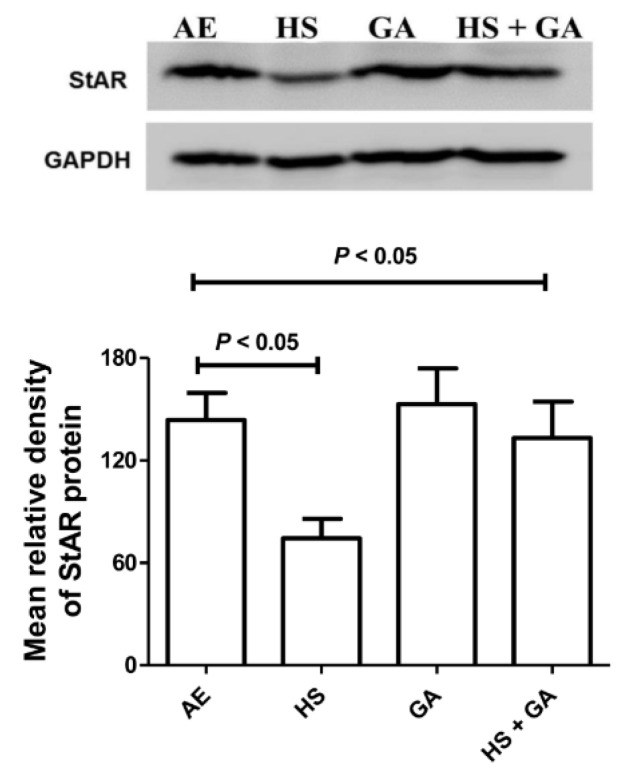
Representative immuno-Western blot of testicular StAR protein in control and hookah smoke (HS)-exposed group, with or without gum acacia (GA) treatment. Glyceraldehyde 3-phosphate dehydrogenase (GAPDH) was used as internal control for the antibodies. The histograms represent the average means ± SEM of relative density of protein bands quantified from three independent experiments using different set of testes in each experiment. There was a significant decrease in the StAR protein in the tissue protein homogenate isolated from testes of mice exposed to HS, an action that was significantly reversed by GA concomitant treatment.

Western blot analysis showed that the level of testicular steroidogenic acute regulatory protein (StAR) was significantly reduced in mice exposed to HS, when compared with the control mice group exposed to normal air. This action was significantly reversed when mice were exposed to HS and treated simultaneously with GA (Figure 8).

## 4. Discussion

In this work, we showed that exposure of mice to HS for 30 consecutive days caused significant reductions in the testicular protective antioxidant and anti-inflammatory indices, and in the plasma concentrations of the hormones testosterone, estradiol, and LH. This confirms that the exposure of male mice to HS for 30 consecutive days, which causes several adverse effects in the respiratory and cardiovascular systems [21,25], can also have deleterious effect on the reproductive system of male mice. The present study also confirmed the previously reported finding about the presence of slight impairment of spermatogenesis, as seen in the light microscopic testicular histology in mice exposed to HS [23]. However, more pronounced testicular histological damage was seen after exposure of HS for a longer duration, viz., six months [12].

There are conflicting reports on the effect of exposure of humans to CS on reproductive hormones. However, a relatively recent review [26] showed that, in general, CS has a negative impact on reproduction, including reproductive hormones, ascribed mainly to tobacco alkaloids (nicotine and its metabolite cotinine). More recently, it has been reported that nicotine, as well as other cigarette toxins, also dysregulate the reproductive and hormonal system [27]. As far as we are aware, no similar studies have been conducted on HS in either humans or animals, apart from a few publications [12,23]. Results obtained from the present work confirm the deleterious effect of exposure to HS on the reproductive hormones in male mice, even for a relatively short duration of 30 days. Furthermore, we have shown that co-administration of GA mitigated significantly these deleterious actions, probably through amelioration of the adverse actions of HS (viz., inflammation, oxidative and nitrosative stress, and other biochemical actions).

Nitrosative and oxidative stresses were found to be involved in the general inflammatory response that occurs because of metabolic imbalance caused by excessive generation of reactive nitrogen and oxygen species (RNS and ROS, respectively) and/or a decreased action of host antioxidant defense mechanisms [28]. During inflammation, oxygen is utilized at a high rate, releasing mitochondrial superoxide free radical, which, in turn, impairs mitochondrial function, leading to cell and organ dysfunction. Therefore, antioxidants can remove excess ROS and protect the integrity of cells. Oxidative stress also potentiates the associated inflammatory reactions by activating some different pathways [28]. Tobacco smoke has been found to induce oxidative and nitrosative stress, and inflammation in both humans and animals [29,30]. More recently, tobacco smoke has also been shown to induce nitrosative stress that can lead to some pathological condition in smokers [31]. It is known that more than 5000 chemicals are present in tobacco smoke, many of which are toxic and carcinogenic [32]. It is not known with certainty which of these chemicals are responsible for inducing inflammation and oxidative and nitrosative stress in smokers. However, more is known about the role of nicotine in oxidative and nitrosative stress, and inflammation induced by tobacco smoke [33]. Nicotine (0.5 and 1 mg/kg), when orally administered to rats for 30 days, has been reported to significantly decrease several indices of the antioxidant profile of plasma of rats [34]. In this work the oxidative action of HS and the antioxidative action of GA on the seminiferous tubules were confirmed biochemically and immunohistochemically (using SOD as an index). Oxidative damage is linked to the disruption in the mitochondria of mouse (MA-10) tumor leydig cells, and reduce the expression of steroidogenic acute regulatory protein (StAR), which in turn inhibits steroidogenesis [35]. HS exerted oxidative damage in mice testes, resulting in significant decrease in StAR protein levels. Gum acacia (GA) is known for its antioxidant effect [17], and when given to mice simultaneously exposed to HS, it enhanced the defense against the ROS, and antagonized the decrease in testicular StAR. Interestingly, GA has been reported to improve the quality of the semen and abrogate oxidative stress in diabetic rats [36].

The present work showed that concomitant administration of GA significantly mitigated all of the measured indices of oxidative and nitrosative stress, and inflammation in the testicular homogenates from mice exposed to HS. GA has been shown to possess antioxidant and anti-inflammatory properties in rats and humans [16,17,20,37]. Recently, it has been confirmed that consumption of agents with anti-inflammatory and antioxidant properties is beneficial to the quality of sperm, and improves progeny survival in the zebrafish [38].

Our study concluded that daily exposure of male mice for 30 days to HS caused some adverse actions on the reproductive hormones levels in plasma and indices of oxidative and nitrosative stress and inflammation in the testes, and GA treatment alleviated these effects by reducing the inflammation and oxidative and nitrosative stress, via a mechanism involving Nrf2, and reduction of the expression of the steroidogenic acute regulatory protein StAR.

A study of the functional consequences of HS exposure (and effect of GA thereon) on male reproduction is warranted. Depending on the results of such a study, and pending further pharmacological and toxicological studies, intake of GA supplement may be recommended as a useful agent that can mitigate the adverse effect of tobacco use.

## Figures and Tables

**Table 1:** Some physiological parameters of mice exposed to air (AE) or hookah smoke (HS) with or without gum acacia (GA) treatment.

Parameters/Treatment	AE	HS	GA	HS + GA
Initial Body weight (g)	21.4 ± 0.6	21.3 ± 0.62	20.8 ± 0.56	25.4 ± 0.65
Final Body weight (g)	22.9 ± 0.38	20.6 ± 0.56	20.6 ± 0.63	22.5 ± 0.42
Body weight change (%)	3.3 ± 2.5	−2.8 ± 1.9	−0.6 ± 1.2	1.0 ± 2.4

Reported values are mean ± standard error of the mean (SEM) (*n* = 8). GA (15% *w*/*v*) was given to mice in the drinking water for 30 days, during which they were exposed to HS (30 min/day for 30 days).

**Table 2:** Some plasma parameters in mice exposed to air (AE) or hookah smoke (HS) with or without GA treatment.

Parameters/Treatment	AE	HS	GA	HS + GA
Testosterone (ng/mL)	5.14 ± 0.3	2.0 ± 0.2 ^a^	5.2 ± 0.5	4.1 ± 0.2 ^b^
Estradiol (pg/mL)	28.57 ± 5.9	10.83 ± 0.6 ^a^	34.3 ± 2.58	17.8 ± 0.8 ^c^
LH (mIU/mL)	37.4 ± 1.2	28.2 ± 0.9 ^a^	41.0 ± 1.5	30.9 ± 1.0 ^b,c^
Inhibin B (pg/mL)	9.24 ± 0.71	66.45 ± 6.26 ^a^	6.44 ± 1.68	37.51 ± 6.22 ^b,c^
ABP (ng/mL)	9.67 ± 0.51	5.39 ± 0.51 ^a^	9.73 ± 0.96	8.32 ± 0.56 ^b^

Reported values are mean ± SEM (*n* = 8). GA (15% *w*/*v*) was given to mice in the drinking water for 30 days, during which they were exposed to HS (30 min/day for 30 days). LH (luteinizing hormone), ABP (androgen binding protein). Different superscripts indicate significance as follows (*p* < 0.05 was considered significant): ^a^ Indicates significance of control group vs. different groups, ^b^ indicates significance of HS group vs. (HS + GA)-treated group, ^c^ indicates significance of GA group vs. (HS + GA)-treated group.

**Table 3:** Urine cotinine level of mice exposed to air (AE) or hookah smoke (HS) with or without GA treatment.

Group	Cotinine (ng/mL)
**AE**	1.19 ± 0.13
**HS**	3.05 ± 0.6 ^a^
**GA**	1.11 ± 0.07
**GA + HS**	1.29 ± 0.09 ^b^

The values reported are mean ± SEM (*n* = 8). GA (15% *w*/*v*) was given to mice in the drinking water for 30 days, during which they were exposed to HS (30 min/day for 30 days). Different superscripts indicate significance as follows (*p* < 0.05 was considered significant): **^a^** Indicates significance of control group vs. different groups, **^b^** indicates significance of HS group vs. (HS + GA)-treated group.

**Table 4:** Johnsen’s mean testicular biopsy score in each experimental groups.

Johnsen Score %										
	10	9	8	7	6	5	4	3	2	1
AE	51	17	20	7	3	2	0	0	0	0
HS	39	17	21	4	4	15	0	0	0	0
GA	64	13	10	7	4	2	0	0	0	0
HS + GA	59	12	17	7	4	1	0	0	0	0

Values in the table are represented as % of the seminiferous tubules which achieved a particular Johnsen’s score in the air exposure (AE), hookah smoke (HS), gum acacia (GA), and HS + GA groups (*n* = 5–6). GA (15% in water) was given to the mice in the drinking water for 30 days, concomitantly with either HS or AE.
